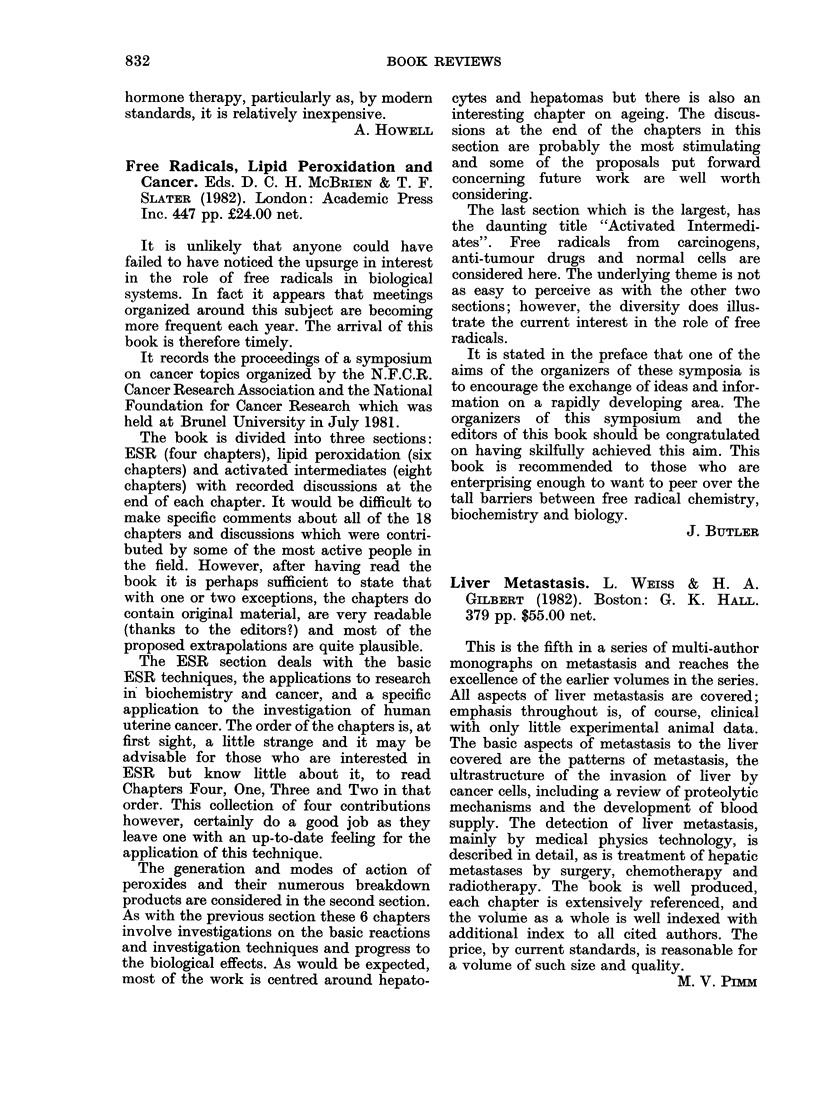# Free Radicals, Lipid Peroxidation and Cancer

**Published:** 1982-11

**Authors:** J. Butler


**Free Radicals, Lipid Peroxidation and Cancer.** Eds. D. C. H. McBrien & T. F. Slater (1982). London: Academic Press Inc. 447 pp. £24.00 net.

It is unlikely that anyone could have failed to have noticed the upsurge in interest in the role of free radicals in biological systems. In fact it appears that meetings organized around this subject are becoming more frequent each year. The arrival of this book is therefore timely.

It records the proceedings of a symposium on cancer topics organized by the N.F.C.R. Cancer Research Association and the National Foundation for Cancer Research which was held at Brunel University in July 1981.

The book is divided into three sections: ESR (four chapters), lipid peroxidation (six chapters) and activated intermediates (eight chapters) with recorded discussions at the end of each chapter. It would be difficult to make specific comments about all of the 18 chapters and discussions which were contributed by some of the most active people in the field. However, after having read the book it is perhaps sufficient to state that with one or two exceptions, the chapters do contain original material, are very readable (thanks to the editors?) and most of the proposed extrapolations are quite plausible.

The ESR section deals with the basic ESR techniques, the applications to research in biochemistry and cancer, and a specific application to the investigation of human uterine cancer. The order of the chapters is, at first sight, a little strange and it may be advisable for those who are interested in ESR but know little about it, to read Chapters Four, One, Three and Two in that order. This collection of four contributions however, certainly do a good job as they leave one with an up-to-date feeling for the application of this technique.

The generation and modes of action of peroxides and their numerous breakdown products are considered in the second section. As with the previous section these 6 chapters involve investigations on the basic reactions and investigation techniques and progress to the biological effects. As would be expected, most of the work is centred around hepatocytes and hepatomas but there is also an interesting chapter on ageing. The discussions at the end of the chapters in this section are probably the most stimulating and some of the proposals put forward concerning future work are well worth considering.

The last section which is the largest, has the daunting title "Activated Intermediates". Free radicals from carcinogens, anti-tumour drugs and normal cells are considered here. The underlying theme is not as easy to perceive as with the other two sections; however, the diversity does illustrate the current interest in the role of free radicals.

It is stated in the preface that one of the aims of the organizers of these symposia is to encourage the exchange of ideas and information on a rapidly developing area. The organizers of this symposium and the editors of this book should be congratulated on having skilfully achieved this aim. This book is recommended to those who are enterprising enough to want to peer over the tall barriers between free radical chemistry, biochemistry and biology.

J. Butler